# Case Report: IBD-like colitis following CAR T cell therapy for diffuse large B cell lymphoma

**DOI:** 10.3389/fonc.2023.1149450

**Published:** 2023-05-22

**Authors:** Sebastian Zundler, Francesco Vitali, Soraya Kharboutli, Simon Völkl, Iris Polifka, Andreas Mackensen, Raja Atreya, Markus F. Neurath, Dimitrios Mougiakakos

**Affiliations:** ^1^ Department of Medicine 1 – Gastroenterology, Pneumology, Endocrinology, University Hospital Erlangen, Erlangen, Germany; ^2^ University Hospital Erlangen, Deutsches Zentrum Immuntherapie, Erlangen, Germany; ^3^ Department of Medicine 5 – Hematology/Oncology, University Hospital Erlangen, Erlangen, Germany; ^4^ Institute of Pathology, University Hospital Erlangen, Erlangen, Germany; ^5^ Department for Hematology and Oncology, University Hospital Magdeburg, Magdeburg, Germany

**Keywords:** diffuse large B cell lymphoma, inflammatory bowel disease (IBD), tisagenlecleucel, case report, CAR T cells

## Abstract

Chimeric antigen receptor (CAR) T cell therapy has become a new mainstay in the treatment of several hematologic malignancies, but the spectrum of associated complications is still incompletely defined. Here, we report the case of a 70-year-old female patient treated with tisagenlecleucel for diffuse large B cell lymphoma (DLBCL), who developed chronic diarrhea with characteristics of inflammatory bowel disease (IBD)-like colitis. CAR T cells were substantially enriched in the colon lamina propria and other diagnoses were ruled out. Thus, we conclude that IBD-like colitis in this patient was associated to CAR T cell therapy and needs to be considered as a rare potential complication.

## Case report

Chimeric antigen receptor (CAR) T cell therapy has become a new mainstay in the treatment of several hematologic malignancies (1). However, the spectrum of complications associated with CAR T cell therapy is still incompletely defined (2).

We describe the case of a 70-year-old female patient of Turkish origin with a past history of diffuse large B cell lymphoma (DLBCL) treated with a commercial anti-CD19 CAR T-cell product (i.e., tisagenlecleucel), who presented with chronic diarrhea.

Initially, the patient had been diagnosed with follicular lymphoma in 2007 and received chemotherapy with R-CHOP, R-Bendamustin, R-Gemcitabin/Oxaliplatin and idelalisib over the following years. Transformation into an aggressive B cell non-Hodgkin lymphoma was first noted in 2014 and treated with chemotherapy combining ifosfamid, carboplatin and etoposide followed by high-dose chemotherapy (HDT) with autologous stem cell transplantation (ASCT), R-Revlimid and radiation.

In September 2020, imaging noted a relapse with vertebral and paravertebral involvement. Histology from a punch biopsy as well as on tissue obtained during neurosurgical intervention led to the diagnosis of DLBCL and CAR T cell therapy was performed in November 2020.

**Figure 1:**
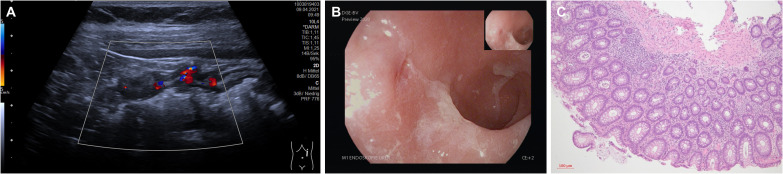
**(A)** Findings on bowel ultrasound (proximal descending colon). **(B)** Findings on colonoscopy (sigmoid colon) **(C)** Findings on histopathology (H/E staining, 10x magnification).

Three months after CAR T-cell therapy and being in complete metabolic remission (CMR), the patient reported the development of up to 20 loose stools per day. Over the following weeks, symptoms persisted, and the patient noticed unintentional weight loss. Stool cultures for pathogenic bacteria were repetitiously negative. Similarly, norovirus, astrovirus, and cytomegalovirus infection were ruled out. On ultrasound, we noted a discontinuous pancolitis with bowel wall thickening and hyperemia (**Figure 1A**). A subsequent colonoscopy confirmed discontinuous pancolitis with redness, swelling, fibrin exudates, and erosions (**Figure 1B**). Previous FDG-PET scan had not shown any metabolic activity in the colon and, accordingly, histopathologic evaluation ruled out DLBCL infiltration. However, it revealed signs of chronic inflammation of the colon mucosa with crypt distortions and some crypt abscesses (**Figure 1C**). The patient did not take any non-steroidal anti-inflammatory drugs and there was no indication of atherosclerosis.

Together, these diagnostic results demonstrated presence of an inflammatory bowel disease (IBD). Yet, given the disease and patient history, neither Crohn’s disease nor ulcerative colitis were likely.

**Figure 2:**
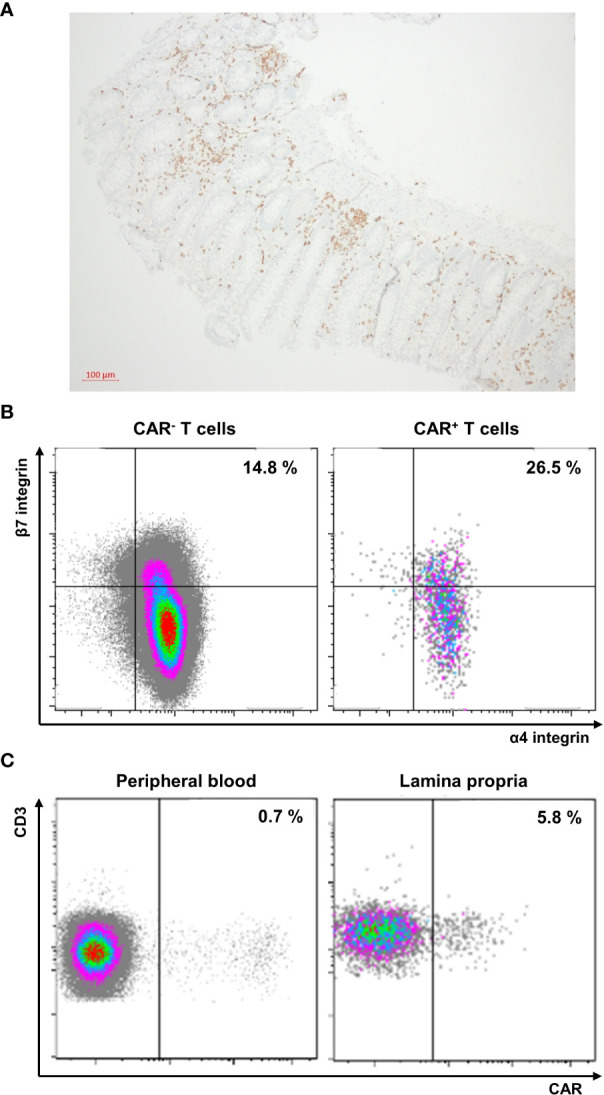
**(A)** Immunohistochemistry of a colon biopsy for CD3 (10x magnification). **(B)** Flow cytometry of peripheral blood T cells expressing the chimeric antigen receptor (CAR) or not. The frequency of α4^+^β7^+^ cells is indicated. CAR T cells were analyzed as previously described (3). Briefly, peripheral blood mononuclear cells were isolated by density centrifugation, stained with CD19 CAR detection reagent, washed twice and stained with Biotin antibody (both Miltenyi Biotec, Bergisch-Gladbach, Germany), 7-AAD (BD Biosciences) and a standardized panel of antibodies against CD45, CD3, CD4, CD8 (all BD Biosciences, Heidelberg, Germany), α4 integrin and β7 integrin (both Biolegend). Gating strategies are depicted in the **Supplementary Figure 1**. Data were acquired on a LSRFortessa (BD Biosciences) and analyzed by Kaluza software v2.1 (Beckman Coulter, Krefeld, Germany). **(C)** Flow cytometry of peripheral blood (left) and lamina propria (right) T cells. The frequency of CAR^+^ cells is indicated.

Further work-up of colon biopsies with immunohistochemistry for CD3 demonstrated a marked enrichment of T cells in the intestinal lamina propria (**Figure 2A**). Flow cytometry of CAR^+^ and CAR^-^ T cells in the peripheral blood showed that a subset of the CAR T cells expresses the gut-homing receptor α4β7 and that α4β7-expressing cells are enriched within CAR^+^ compared to CAR^-^ T cells (**Figure 2B**, **Supplementary Figure 1**). Consistently, we observed a substantial enrichment of CAR T cells in the colon lamina propria (**Figure 2C**).

A course of prednisolone starting with 50 mg per day was initiated (iv over the first ten days, then orally) and tapered over seven weeks, but no symptomatic improvement was seen. The patient was subsequently scheduled for treatment with the anti-α4β7 integrin antibody vedolizumab. However, this was not initiated, since ultimately before the first planned application and more than four months after symptom onset, diarrhea spontaneously and gradually resolved, and no signs of colitis were detected on follow-up ultrasound. There was no correlation of diarrhea with CAR^+^ T cell frequencies in the peripheral blood (**Supplementary Figure 2**) and, interestingly, resolution occurred following a two-week course of antibiotic treatment with empiric levofloxacin plus vancomycin followed by linezolid for bacteremia due to central venous line infection.

## Discussion

We conclude that IBD-like colitis in this patient was triggered by CAR T cells. CAR T cell therapy has revolutionized the treatment of several hematologic malignancies (4–6). Tisagenlecleucel comprises expanded autologous T cells engineered with a CAR to target CD19. Here, it seems that the *ex vivo* expansion of certain gut-homing T cell clones during CAR T cell production led to the recruitment of large numbers of these cells to the large bowel following adoptive transfer. We speculate that their interaction with the intestinal microbiome *via* the original T cell receptor may have triggered local expansion and IBD-like inflammation in this patient (7). Eventually, resolution of colitis following an episode of antibiotic therapy even highlights the possibility that a change in the intestinal microbiota might have limited further stimulation of these intestinal CAR T cell clones.

The trials leading to approval of anti-CD19 CAR T cells were relatively small (6) and, thus, previously unknown adverse events are continuing to emerge. The most common specific side effects of CAR T cell therapy described to date are the cytokine release syndrome (CRS) and the immune effector cell associated neurotoxicity syndrome (ICANS) (8, 9). CRS is triggered by activation of CAR T cells following target antigen recognition, which in turn stimulate (by e.g., abundant GM-CSF, IFN-γ or TNF) the myeloid compartment that releases high levels of IL-6. This activation of myeloid cells can aggravate to macrophage activation syndrome (MAS) or hemophagocytic lymphohistiocytosis (HLH), which are very severe pathologies. Consequently, first-line therapy consists of blocking the IL-6 receptor. In case of therapy failure, primarily steroids are used, but also antibodies against TNF, IL-1 or tyrosine kinase inhibitors can be used, especially in clinically frustrating situations. The pathophysiology of ICANS is less well understood. It can take place with or without a CRS and the clinical picture is also very inhomogeneous. Vascular permeability, endothelial disruption, and glial cell injury appear to be involved in the clinical picture, and the primary treatment of isolated ICANS is steroid therapy. Another bothersome side effect, which only became apparent as the number of CAR T cell-treated patients increased, was prolonged hematotoxicity. The cause of this phenomenon is not conclusively understood, but an inflammatory milieu appears to be an important trigger (10).

These side effects are in significant part due to overactivation of CAR T cells. Many concepts are being evaluated in this regard, ranging from the preventive administration of IL-6 receptor antagonists to the genetic modification of CAR T cells. Here, the installation of “off-switches”, the better adjustment of the signal strength by modification of the affinity to the target antigen or the costimulatory domain and also the suppression of cytokine release are conceivable (11).

Collectively, these adverse events are in one or another way linked to the function of the CAR T cells. However, that also the original T cell receptor (TCR) of the expanded clonotypes might cause side effects has not been acknowledged so far. Such a risk could be avoided by genetic removal of the endogenous TCR as it already occurs during generation of allogeneic CAR T cells (12). However, it is important to consider here that the endogenous TCR also seems to be important for the persistence of CAR T cells (13). Overall, we consider the probability of transduction of T cells carrying an autoreactive TCR with the CAR construct to be very low. However, it cannot be ruled out and should at least be considered, especially in cases of organ-specific complications (beyond generalized inflammation).

In conclusion, this case is the first to report IBD-like CAR T cell-associated colitis following therapy with tisagenlecleucel. Moreover, while a previous report described similar symptoms following therapy with axicabtagene ciloleucel (14), it is also the first to directly demonstrate the presence of CAR T cells in the inflamed intestinal mucosa.

Thus, IBD-like colitis seems to be a rare side effect that should be considered in the differential diagnosis in patients with chronic colitis following CAR T cell therapy.

## Data availability statement

The original contributions presented in the study are included in the article/**Supplementary Material**. Further inquiries can be directed to the corresponding author.

## Ethics statement

Ethical review and approval was not required for the study on human participants in accordance with the local legislation and institutional requirements. The patients/participants provided their written informed consent to participate in this study. Written informed consent was obtained from the participant/patient(s) for the publication of this case report.

## Author contributions

SZ: ultrasound, endoscopy, data integration and analysis, drafting of the manuscript. FV: endoscopy. SK: outpatient care. SV: flow cytometry. IP: histology. AM: supervision of diagnostic and therapeutic management. RA: IBD consultation. MN: supervision of diagnostic and therapeutic management. DM: outpatient care, data integration and analysis. All authors contributed to the article and approved the submitted version.

## Supplementary material

Supplementary Figure 1 Gating strategy for CAR detection: Time parameter was used to monitor instrument stability, doublets were excluded by FSC-H/FSC-A, CD45^+^ events were gated, lymphocytes were determined by FSC-A/SSC-A, viable T cells were gated by CD3^+^ and 7-AAD^-^, and further subdivided in CAR^+^ and CAR^-^ T cells.

Supplementary Figure 2 Time course of frequencies (left panel) and numbers (right panel) of CAR^+^ T cells in the peripheral blood of the patient as determined by flow cytometry.
