# Supplementary figures and images for: Case Report: IBD-like colitis following CAR T cell therapy for diffuse large B cell lymphoma
Source: Front Oncol. 2023 May 22;13:1149450. doi: 10.3389/fonc.2023.1149450 (PMC10240064; doi:10.3389/fonc.2023.1149450)

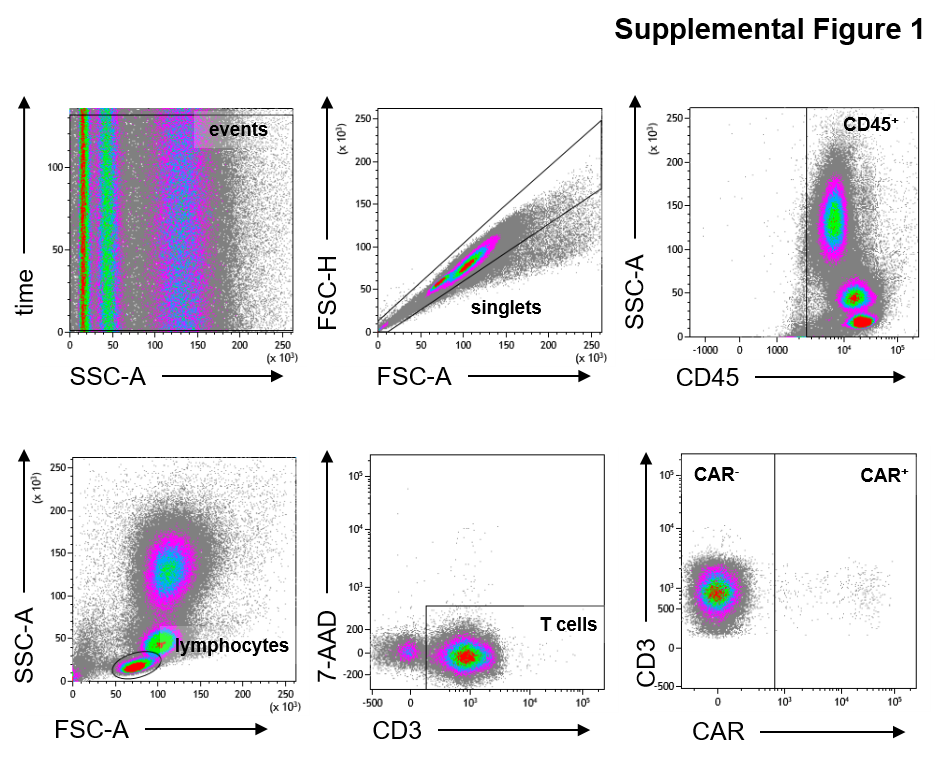

Supplement: Supplementary Figure 1 — Gating strategy for CAR detection: Time parameter was used to monitor instrument stability, doublets were excluded by FSC-H/FSC-A, CD45+ events were gated, lymphocytes were determined by FSC-A/SSC-A, viable T cells were gated by CD3+ and 7-AAD-, and further subdivided in CAR+ and CAR- T cells. [file Image_1.tif]

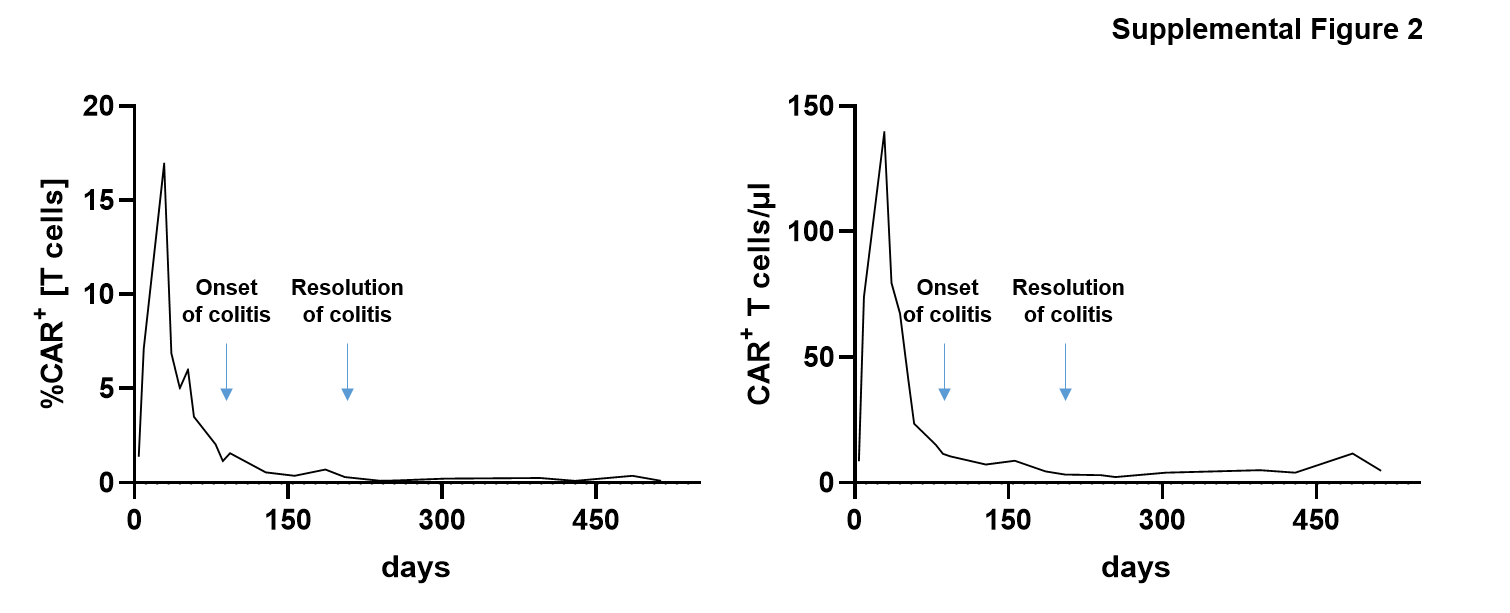

Supplement: Supplementary Figure 2 — Time course of frequencies (left panel) and numbers (right panel) of CAR+ T cells in the peripheral blood of the patient as determined by flow cytometry. [file Image_2.tif]
